# Fat-to-heart crosstalk in health and disease

**DOI:** 10.3389/fgene.2023.990155

**Published:** 2023-03-24

**Authors:** Fleur Lodewijks, Timothy A. McKinsey, Emma L. Robinson

**Affiliations:** ^1^ Department of Pathology, Faculty of Health, Medicine and Life Sciences, Maastricht University, Maastricht, Netherlands; ^2^ Department of Medicine, Division of Cardiology and Consortium for Fibrosis Research and Translation, University of Colorado Anschutz Medical Campus, Aurora, CO, United States

**Keywords:** cardiovascular disease, crosstalk, adipose tissue, heart failure, inflammation, microRNAs

## Abstract

According to the latest World Health Organization statistics, cardiovascular disease (CVD) is one of the leading causes of death globally. Due to the rise in the prevalence of major risk factors, such as diabetes mellitus and obesity, the burden of CVD is expected to worsen in the decades to come. Whilst obesity is a major and consistent risk factor for CVD, the underlying pathological molecular communication between peripheral fat depots and the heart remains poorly understood. Adipose tissue (AT) is a major endocrine organ in the human body, with composite cells producing and secreting hormones, cytokines, and non-coding RNAs into the circulation to alter the phenotype of multiple organs, including the heart. Epicardial AT (EAT) is an AT deposit that is in direct contact with the myocardium and can therefore influence cardiac function through both mechanical and molecular means. Moreover, resident and recruited immune cells comprise an important adipose cell type, which can create a pro-inflammatory environment in the context of obesity, potentially contributing to systemic inflammation and cardiomyopathies. New mechanisms of fat-to-heart crosstalk, including those governed by non-coding RNAs and extracellular vesicles, are being investigated to deepen the understanding of this highly common risk factor. In this review, molecular crosstalk between AT and the heart will be discussed, with a focus on endocrine and paracrine signaling, immune cells, inflammatory cytokines, and inter-organ communication through non-coding RNAs.

## 1 Introduction

Cardiovascular disease (CVD) is currently the world’s leading cause of death. CVDs encompass both disorders of the heart and vasculature, including heart failure (HF) and atherosclerosis. According to data provided by the World Health Organization (WHO), 17.9 million people died from CVD in 2016 (WHO, 2021a), with epidemiological studies suggesting that a downward trend is not likely to occur in the near future. One study published in 2018 revealed that 12.1% of the U.S. adult population is diagnosed with at least one CVD in their lifetime (Cardiovascular, 2020). In another study, the prevalence of CVDs in the US adult population was projected to be 40.5% by 2030 (Heidenreich et al., 2011). Multiple reasons could explain this expected sharp increase. Obesity, high adiposity, and diabetes mellitus (DM) are among the highest and most consistent risk factors for CVDs, including HF (Gutierrez et al., 2018). The prevalence of these risk factors is rising steadily worldwide due to the progressive adoption of a “western” lifestyle (Agha and Agha, 2017). In 2016, 13% of all adults worldwide were obese, with the prevalence of obesity having almost tripled between 1975 and 2016 (WHO, 2021b). These statistics are in line with the expected increase in obesity-associated CVDs.

Research is still needed to determine and understand how a greater abundance of adipose tissue (AT) increases the risk of the development of CVDs. AT is not an inert lipid mass but a cellularly heterogeneous and metabolically active tissue, functioning as an endocrine and paracrine organ. Secretory products of AT include hormones, inflammatory cytokines, and other molecular entities such as microRNAs, which have the potential to signal to and influence the cardiac muscle (Kershaw and Flier, 2004). A large storage space for AT is the abdomen, where visceral adipose tissue (VAT) deposits reside in humans. Excess VAT poses a great threat to cardiovascular health (Després, 2012). It surrounds the abdominal organs and exhibits its influence not only on them but also on the heart and the blood vessels (Romero-Corral et al., 2010). Subcutaneous adipose tissue (SAT) lies directly beneath the skin and has different thicknesses in different skin areas. There is some evidence that SAT does not influence cardiac function as much as VAT, as it is less metabolically active and secretes less products (Ronquillo et al., 2019).

**FIGURE 1:**
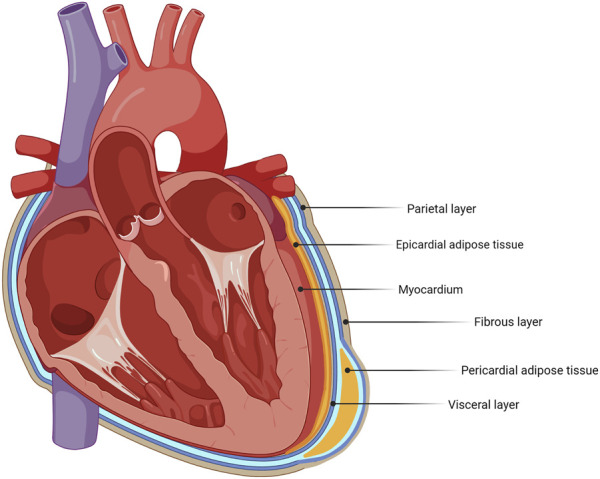
Schematic identifying adipose tissue deposits around the heart. The heart has different adipose tissue deposits, with epicardial adipose tissue (EAT) and pericardial adipose tissue (PAT) as its main deposits.

A major current area of translational research interest is the influence of fat deposits around the heart on cardiac function (Salazar et al., 2016). There is some discrepancy in the literature when addressing the nomenclature of different deposits in the vicinity of the heart. In this review, the following distinction is made, as indicated in Figure 1. Epicardial adipose tissue (EAT) is defined as being directly in contact with the myocardium, without a visceral layer separating them. Pericardial adipose tissue (PAT) lies outside the visceral pericardium and on the external surface of the parietal pericardium (Figure 1). Lipid droplets are also present within cardiac cells and play important roles in cardiomyocyte signaling and immune protection in health, whereas excess intracellular triglyceride deposition can cause lipotoxicity (Jebessa et al., 2019; Monson et al., 2021).

Unanswered questions remain as to how AT influences the risk and development of CVDs. This review will discuss what is known about fat–heart crosstalk in the context of health and disease and, in particular, HF. An overview of the key recent (published within the last 20 years) articles describing molecular mechanisms of crosstalk in the fat–heart axis are summarized in Table 1. Research describing the influence of high adiposity and hyperlipidemia on blood vessels will not be extensively discussed here.

**TABLE 1:** Overview of the literature describing the molecular basis of fat–heart crosstalk.

Signaling	Molecule	Function	Reference
Endocrine	Adiponectin	Cardioprotective adipokine secreted from mature adipocytes with anti-inflammatory properties and influence in fat and glucose metabolism and weight loss	Antonopoulos et al. (2016)
			Mori et al. (2019)
			Wang et al. (2009)
			Bobbert et al. (2011)
			Khan et al. (2012)
			Baltrūnienė et al. (2017)
	Omentin	Cardioprotective adipokine secreted from epicardial adipose tissue. Improves damaging conditions on cardiomyocytes by improving contractility and insulin sensitivity	Greulich et al. (2013)
			Zhou et al. (2020) (Chen et al., 2020)
			Chen et al (2021) (Zhou et al., 2020)
	Vaspin	Cardioprotective adipokine with antiapoptotic effects by activating the PI3K/AKT pathway	Ke et al. (2018)
			Li et al. (2019)
			Rui et al. (2020)
			Zhang et al. (2021b)
	Resistin	Pathogenic adipokine with the ability to alter cardiac remodeling and decrease contractility	Patel et al. (2003)
			Akoumianakis and Antoniades (2017)
			Smith et al. (2011)
			Butler et al. (2009)
			Zhao et al. (2021)
			Silswal et al. (2005)
			Park and Ahima (2013)
	Activin A	Pathogenic adipofibrokine with influence on multiple cardiomyopathies, like HF and cardiac apoptosis	Zeller et al. (2019)
			Venteclef et al. (2015)
			Dani (2013)
			Blumensatt et al. (2013)
			Zaragosi et al. (2010)
	Leptin	Pro-hypertrophic in isolated neonatal rat ventricular cardiac myocytes	Rajapurohitam et al. (2003)
		Suppression of pro-inflammatory mediators and in an *in vivo* model of cardiac inflammation (LPS)	Xu et al. (2004)
			Abd Alkhaleq et al. (2021)
	Apelin	Administration of apelin in a mouse model of chronic high-fat feeding improved left ventricular hypertrophy, fibrosis, systolic, and diastolic function and cardiomyocyte calcium handling	Ceylan-Isik et al. (2013)
Micro RNA	miR-130b-3p	Suppression of multiple anti-apoptotic/cardioprotective molecules in cardiomyocytes induces damage leading to apoptotic cardiac cells	Gan et al. (2020)
	miR-223-3p	Promotes inflammatory environment by attracting macrophages and induced change to M1 phenotype. Cardiac hypertrophy could be induced and could lead to HF	Sánchez-Ceinos et al. (2021)
			Zhang et al. (2021a)
	miR-143	Inhibits the activation of the Akt pathway regulating glucose uptake by insulin through the downregulation of a novel regulator of insulin action, ORP8	Blumensatt et al. (2013)
			Zaragosi et al. (2010)
			Ogawa et al. (2020)
	miR-29a	Can be either protective or pathogenic to the myocardium. Inhibits the Akt/mTOR pathway that can lead to HF and hypertrophy but can also protect against both myopathies	Liu et al. (2019)
			Caravia et al. (2018)
			Shiyu et al. (2019)
			Liu et al. (2020)
			Zhang et al. (2019)
Inflammation	TNF-α	Pro-inflammatory cytokine	Liddle et al. (2020)
			Schumacher and Naga Prasad (2018)
	IL-6	Cytokine with both pro- and anti-inflammatory properties	Whitham et al. (2019)
			Jones and Jenkins (2018)
			Sindhu et al. (2015)
			Han et al. (2020)
			Chen et al. (2022)
	IL-8	Cytokine pro-inflammatory	Damås et al. (2000)
			Nymo et al. (2014)
	Macrophages	Immune cell with multiple roles like cytokine secretion and phagocytosis. Accumulated in obesity	Suganami et al. (2005)
			Lumeng et al. (2007)
	CD8^+^ cells	Adaptive immune cell that is cytotoxic and recruits macrophages in early stages in obesity	Nishimura et al. (2009)
			Yang et al. (2010)
	NLRP3 inflammasome	Assembled structure of multiple components that cleaves pre-IL-1beta to its active form	Wang et al. (2020)
			Koenen et al. (2011)
			Sokolova et al. (2019)
			Vandanmagsar et al. (2011)

## 2 MicroRNAs

**FIGURE 2:**
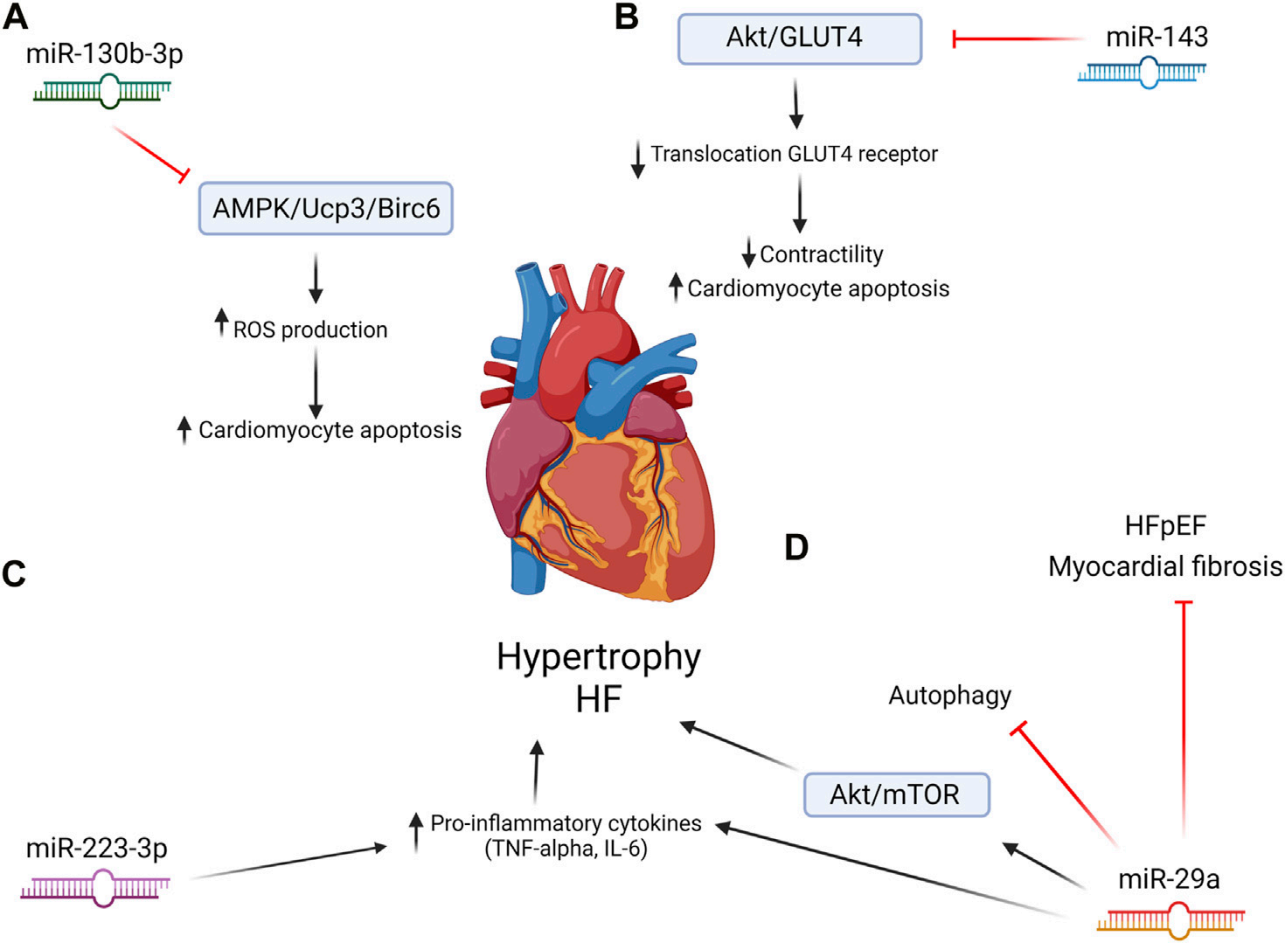
AT-derived microRNAs and their impacts on heart failure and cardiac hypertrophy. **(A)** miR-130b-3p inhibits the AMPK/Ucp3/Birc6 pathway, which increases ROS production and cardiomyocyte damage. **(B)** miR-143 inhibits the Akt/GLUT4 pathway, resulting in decreased contractility and cardiomyocyte apoptosis. **(C)** miR-223-3p induces increased pro-inflammatory cytokine production. **(D)** mir-29a directly and indirectly promotes heart failure but can also be cardioprotective.

An increasing body of evidence supports the importance of microRNAs (miRNAs) in the pathobiology of CVDs. miRNAs are 18–21 bp single-stranded non-coding RNAs that act by silencing RNA through post-transcriptional regulation of gene expression. By binding to complementary RNA sequences in coding or long non-coding RNAs, miRNAs can silence translation and/or destabilize and promote the degradation of target RNAs (Bartel, 2018; O’Brien et al., 2018). Alterations in levels of miRNAs in adipocytes can influence the myocardium to such extent that it can assume a pathophysiological role by initiating pathological hypertrophy and HF through ultimately promoting a cardio-detrimental proteome (Stepien et al., 2018). A small number of miRNAs have been implicated in crosstalk between AT and the myocardium (Figure 2).

### 2.1 miR-223-3p

miR-223-3p has been reported to contribute to pathophysiological remodeling in the heart by stimulating a pro-inflammatory state in macrophages (Yang et al., 2016). It is produced by both cardiomyocytes and adipocytes (Bartel, 2018; Stepien et al., 2018). miR-223-3p has been reported to contribute to the progression of HF by stimulating a pro-inflammatory state in macrophages. Zhang et al. (2021a) found that the induction of pro-inflammatory cytokine release increases stress on the myocardium, which could lead to HF. Such a pro-inflammatory cytokine produced by M1 macrophages is tumor necrosis factor-alpha (TNF-α), and it was identified as a downstream target regulator by Sánchez-Ceinos et al. (2021). This linked the overexpression of miR-223-3p to an inflammatory environment, which is also present in patients with a high adiposity. However, miR-223-3p was reported to have an inductive effect in cardiac hypertrophy, leading to HF. More *in vivo* studies are needed to further explore their functions in the progression of HF (Zhang et al., 2021a).

Furthermore, miR-223-3p was progressively internalized into adipocytes in the presence of pathophysiological stimuli, including DM and obesity. This internalization was associated with metabolic dysfunction in adipocytes and contributed to compromised signaling pathways, for example, in lipolysis, which could lead to multiple pathologies, including CVD (Sánchez-Ceinos et al., 2021).

### 2.2 Activin A

The adipofibrokine activin A is highly expressed in adipocytes and is associated with proliferation and differentiation of adipocyte progenitors. It signals through type I and type II transmembrane serine/threonine proteases and through SMAD2/3 to bring about responses in effector cells (Abe et al., 2004). It is a critical component in stem cell proliferation and has the ability to induce fibrotic phenotypes in adipocytes, which could lead to pathological outcomes (Zaragosi et al., 2010; Dani, 2013). A study by Zaragosi et al. (2010) reported that in obese patients, activin A is more highly expressed in AT than in lean subjects; it also upregulated macrophage-secreted factors, which has a higher concentration in AT. Excess levels of activin A can lead to multiple pathological phenotypes. First, it can block differentiation of preadipocytes into mature adipocytes. This could be interesting in the treatment of obesity to control the size and phenotype of adipocytes (Dani, 2013). In patients with severe obesity, activin A was significantly related to multiple CVDs and their co-morbidities such as left ventricular diastolic disorder. This study also reported that activin A is present in the secretory profile of EAT and contributes to EAT thickness (Zeller et al., 2019). This is confirmed by Venteclef et al. (2015), who provided evidence that activin A in the secretory profile in EAT can lead to myocardial fibrosis and AF, which in turn can lead to HF. As mentioned before (see Section 2.3), activin A upregulates miR-143, which could lead to cardiomyopathy (Blumensatt et al., 2013). All the aforementioned pathologies and working mechanisms can be reversed by decreasing AT, as it decreases activin A levels and reduces the presence of macrophages, decreasing the inflammatory environment (Zaragosi et al., 2010).

### 2.3 miR-130b-3p

A recent study by Gan et al. (2020) examined the role of miR-130b-3p in adipocytes in the control of pathological communication between AT and the myocardium. Small extracellular vesicles (sEV) aid in this communication by transporting miR-130b-3p from the AT to the myocardium. miR-130b-3p was found to have pro-apoptotic effects on cardiomyocytes, resulting in myocardial ischemia/reperfusion injury and, eventually, HF. The pro-apoptotic action of miR-130b-3p is due to inhibition of a number of targets, including AMPK and Ucp3, inducing reactive oxygen species (ROS) and DNA damage, leading to apoptosis. Inhibition of miR-103b-3p with an antagomiR prevented pathological changes in the myocardium, making miR-130b-3p a potentially interesting therapeutic target (Gan et al., 2020).

### 2.4 miR-143

Activin A is a molecule produced by AT that upregulates the production of miR-143 (Zaragosi et al., 2010; Blumensatt et al., 2013). miR-143 was found to inhibit the Akt/GLUT4 pathway by the translocation of the GLUT receptor to the sarcolemma, reducing contractile functions in cardiomyocytes (Blumensatt et al., 2013). It impairs glucose and insulin uptake by inhibiting ORP8, which is a mediator in insulin action. This is not beneficial as the heart uses glucose for 70% of its needed energy. The upregulation of this molecule could lead to cardiomyocyte death and cardiomyopathy (Blumensatt et al., 2013; Ogawa et al., 2020). A study by Ogawa et al. (2020) demonstrated that upregulated miR-143 expression lead to a dilated cardiomyopathy-like phenotype in cultured cells and transgenic mice. It could induce a reductive redox shift and a sustained activation, which would lead to cardiomyopathy (Ogawa et al., 2020).

### 2.5 miR-29a

Recent studies have implicated miR-29a in multiple co-morbidities of CVDs. miR-29a is expressed in macrophages in VAT in lean and obese mice. In obese mice, miR-29a abundance is elevated compared to that in lean mice. It is packed in exosomes and can migrate into cardiomyocytes and adipocytes (Liu et al., 2019). Since macrophages accumulate in VAT in obesity, this could increase the circulating pool of miR-29a (Weisberg et al., 2003). In rats, elevated miR-29a in the heart activated the AKT/mTOR pathways, which lead to cardiac hypertrophy and eventually HF. It also suppressed autophagy in cardiac cells, leaving damaged cells in the environment, which can contribute to inflammation (Shiyu et al., 2019). Another study found that the overexpression of miR-29a leads to multiple myocardial injuries by the increase in apoptosis and pro-inflammatory cytokines such as interleukin (IL)-6 and TNF-α. When miR-29a was decreased, the damage that the heart sustained was reversed and the production of cytokines was decreased as well (Liu et al., 2020).

However, some studies support a cardioprotective role of miR-29a. Through the regulation of peroxisome proliferator-activated receptor delta (PPARδ), miR-29a was said to reduce cardiac hypertrophy (Liu et al., 2019; Zhang et al., 2019). Another study by Caravia et al. (2018) showed that knockout mice that were deficient in miR-29a developed multiple pathological cardiac features, including myocardial fibrosis and hypertension, that led to HF with preserved ejection fraction (HFpEF). The role of miR-29a in the heart needs further investigation to determine if its role is indeed harmful or cardioprotective when overexpressed as an important regulator of cardiac homeostasis.

## 3 Endocrine, adipokines, and paracrine crosstalk

**FIGURE 3:**
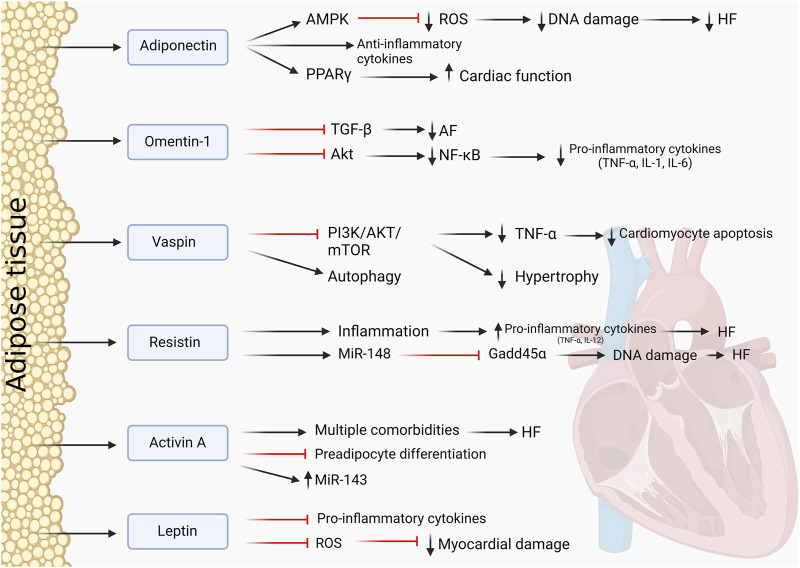
Adipokines and their influence on the heart. Protective and pathological effects of AT-derived adipokines on the cardiac muscle.

AT is a highly active endocrine and paracrine organ, contributing to local, peripheral, and systemic signaling and organ–organ crosstalk, including with the heart. Adipokines are biologically active hormones derived from adipocytes and include adiponectin, resistin, leptin, and cytokines such as IL-6 and TNF-α. Adipokines primarily play a role in the regulation of inflammatory response and insulin resistance. Figure 3 provides an overview of mechanisms of endocrine and paracrine signaling between fat and the heart.

### 3.1 Adiponectin

Adiponectin is a well-established adipokine with cardioprotective properties and extensive crosstalk with the myocardium. It is also thought to be the most abundant of all adipokines and is secreted by AT (Berezin et al., 2020). It is involved in signaling pathways such as PPARγ signaling and induces the AMPK pathway. Through these pathways, adiponectin directly decreased oxidase activity *via* paracrine and endocrine effects, decreasing oxidative stress in the myocardium and protecting the heart from ROS (Antonopoulos et al., 2016). Enhancement of its paracrine effects can be established through the administration of PPARγ agonists, which demonstrated a better cardiac function in rats (Mori et al., 2019). A study by Wang et al. (2009) demonstrated that through AMPK signaling, adiponectin can act as an antioxidant, reducing ROS and exhibiting anti-apoptotic effects. In the context of cardiac inflammation in obesity, adiponectin is upregulated as a protective anti-inflammatory response in an attempt to mitigate cardiac dysfunction (Bobbert et al., 2011). In patients with AT inflammation and HF, adiponectin resistance can occur. This worsened the outcome for patients in the study by Khan et al. (2012), leading to a failing myocardium and HF. This showed that adiponectin is necessary to maintain cardiac health because its absence in cells leads to adverse cardiac effects. Increased levels of adiponectin were found in patients with advanced HF, which could demonstrate that the resistance to adiponectin plays a role in the progression and outcome of an HF patient. Adiponectin can, therefore, also be a biomarker to predict the development and stage of HF and how to progress with treatment options (Baltrūnienė et al., 2017). Adiponectin remains an interesting adipokine to study as it has multiple functions in CVDs and adiposity and the potential to be a good therapeutic agent. A number of clinical trials have assessed the association between circulating adiponectin for risk and prognosis of CVD (e.g., NCT02882672). Administration of AdipoRon, an adiponectin receptor-1 agonist, protects against myocardial injury in ischemia-reperfusion in mice (Zhang et al., 2015). Moreover, adiponectin transgenic overexpressing mice are protected against weight gain, increased adiposity, and adipose inflammation in the context of a high fat–high sucrose diet feeding for 12 weeks (Otabe et al., 2007).

### 3.2 Omentin-1

Omentin-1 is an adipokine expressed primarily by EAT (Greulich et al., 2013). Omentin-1 is reported to have a cardioprotective function, and it is indicated that decreased omentin-1 levels induces cardiovascular dysfunction. Multiple studies have attested to its cardioprotective function, such as the study by Chen et al. (2020). They investigated the role of omentin-1 with regard to AF and atrial fibrosis that could lead to HF. Omentin-1 was demonstrated to be an anti-fibrotic adipokine by inhibiting the transforming growth factor-β (TGF-β) signaling pathways, reversing the effect of AF. It was downregulated in patients with AF, showing that a reduced concentration is indeed pathological to cardiomyocytes (Chen et al., 2020). Another of its protective functions is the suppression of inflammation in AT. In mice that were fed a high-fat diet and had omentin-1 treatment afterward, pro-inflammatory cytokines were reduced, macrophage polarization was reversed, and inflammation was equally reduced (Zhou et al., 2020). In the inflammation signaling, omentin inhibited the Akt pathway, therefore decreasing transcription factor NF-kB. Reduced NF-kB leads to reduced TNF-α secretion and other pro-inflammatory cytokines (Tan et al., 2010). Omentin, therefore, shows potential as a therapy in CVDs to significantly reduce inflammation and reverse pathological conditions, restoring cardiomyocytes toward a healthy state and function.

### 3.3 Vaspin

A more recently discovered adipokine with cardioprotective functions is vaspin. It protects the myocardium by inducing autophagy by inhibiting the PI3K/AKT/mTOR pathway to reduce TNF-α-induced apoptosis (Ke et al., 2018). It also inhibits the NLRP3 inflammasome by inducing autophagy. Vaspin attenuates myocardial injury and aids myocardial repair by continuous inhibition of inflammation (Li et al., 2019). A study by Rui et al. (2020) indicated similar results with vaspin inducing autophagy and, therefore, restricting pathological cardiac hypertrophy by regulating myocardial senescence through inhibition of the PI3K/AKT/mTOR pathway. Most recent literature mentions this signaling pathway as most prominent and promising with regard to vaspin. The cardioprotective properties of vaspin were also examined in mice with lipoatrophy, a loss of inability to form fat tissue, which lead to severe CVDs. Vaspin was administered and resulted in improved cardiac function and repair. Again, vaspin mediated and increased cardiac AKT activity, showing its involvement in the physiological functioning of the heart (Zhang et al., 2021b). Therefore, this could be a potential new target in the field of cardioprotection.

### 3.4 Resistin

Resistin is an adipokine that was previously thought to be expressed and secreted by adipocytes, but the recent literature provides evidence that in humans, resistin is mainly produces by macrophages, lymphocytes, and monocytes within AT (Patel et al., 2003). There is little published work on the effects of resistin in humans, but it is thought to be a cardio-detrimental adipokine (Smith et al., 2011; Akoumianakis and Antoniades, 2017). Circulating levels of resistin were correlated with the risk of developing HF and were shown to be independently associated with this risk (Butler et al., 2009). A study by Zhao et al. (2021) demonstrated with the help of AT-specific resistin, KO-mice that lack resistin showed reduced myocardial fibrosis and improved cardiac function in an HF model. The WT mice showed no improvement, suggesting a role for resistin in promoting pathological remodeling in the heart (Zhao et al., 2021).

Resistin is associated with inflammation, which directly influences cardiomyocytes. Resistin induces secretion of pro-inflammatory cytokines, TNF-α and IL-12, in macrophages (Silswal et al., 2005). Since macrophages are abundant in AT, resistin could influence tissue inflammation in AT and its harmful effects on cardiomyocytes. In mice, studies have found that resistin reduced insulin-simulated glucose uptake and promoted cardiac hypertrophy (Park and Ahima, 2013). Inhibition of resistin could be therapeutically beneficial, suppressing inflammation and adverse cardiac effects. More recently, a further study by Zhao et al. (2022) showed that white adipose tissue-specific resistin knockout mice had attenuated pathological cardiac remodeling in response to transverse aortic constriction-induced pressure overload including lessened cardiomyocyte hypertrophy, fibrosis, and lung congestion. Cardiac overexpression of resistin using AAV-9 technology worsened cardiac dysfunction and myocardial fibrosis. Gene expression data from the hearts of these experimental models suggest that resistin regulates miR148b-3p and Gadd45α in the heart. These data strongly suggest a cardio-detrimental role for resistin.

### 3.5 Leptin

Leptin is a well-characterized adipokine that is involved in regulating metabolism and multiple diseases, such as diabetes type II. Whilst its best studied action is on the brain, where it regulates hunger by communicating energy storage status, leptin receptors have also been found in the heart at a very low level (Green et al., 1995; Münzberg and Morrison, 2015).

Leptin has been found to induce cardiac myocyte hypertrophy in cultured neonatal rat ventricular myocytes along with elevated ROS and MAPK signaling pathways (Rajapurohitam et al., 2003; Xu et al., 2004). This is contradictory to *in vivo* models of HF, where leptin is thought to promote downregulation of pro-inflammatory genes and upregulation of antioxidant genes, resulting in lowered ROS levels and reduced myocardial damage (Abd Alkhaleq et al., 2021). However, a study by Gruzdeva et al. (2022) found that levels of leptin in both EAT and PVAT correlated with similarly elevated levels of IL-6, which lead to increased adipose and systemic inflammation in patients with CAD. Another study showed no difference in leptin levels and influence between HF patients and controls (Karayannis et al., 2013).

In an immortalized murine atrial myocyte cell line, HL-1 cells, exogenous leptin treatment promoted cell proliferation and the ERK/MAPK signaling pathway (Tajmir et al., 2004). It is likely that in HL-1 cells, induction of these signaling pathways is able to promote proliferation rather than hypertrophy, the latter being a more likely response in predominantly post-mitotic primary cardiac myocytes.

There is some evidence for myocardial leptin expression, which may exhibit an autocrine effect on gene expression in the heart. Intramyocardial injection of an antisense oligo against leptin mRNA 5 days post MI significantly reduced IL-1β and IL-6 overexpression and improved cardiac ejection fraction (Moro et al., 2011). However, the cardiac-derived leptin likely contributes minimally to circulating leptin levels in the heart itself.

### 3.6 Other adipokines

Whilst known for its role in the cardiovascular system, the peptide apelin has been found to be expressed in brown AT and has been recognized as an adipokine over the last 15 years (Boucher et al., 2005). Expanded fat depots in obesity in both mice and humans have been associated with higher apelin expression in fat and in the circulation, triggering MAPK and protein kinase C signaling. In a murine model of high-fat feeding for 6 months, administration of apelin by intraperitoneal injection attenuated high-fat diet compromised in left ventricular diastolic and systolic function, and wall thickness, as well as contractile function, and intracellular calcium handling in cardiac myocytes and insulin sensitivity (Ceylan-Isik et al., 2013). Apelin also reduced levels of pro-hypertrophic miRNAs, miR-133a, miR-208, and miR-1 in the heart. The molecular function of apelin as an adipokine on the heart is still emerging.

Circulating levels with a further recently discovered adipokine, chemerin, has been found associated with diabetes, body mass index, circulating triglycerides, and blood pressure (Bozaoglu et al., 2007). However, the chemerin receptor, chemokine-like receptor-1, has not been found on cardiac cells but on cells of vasculature, where it elicits vasoconstriction, thus indirectly increasing pathological pressure on the heart (Kennedy et al., 2016).

Visfatin is an adipokine that has shown potential as a biomarker for the risk of cardiometabolic disease risk. In particular, circulating levels have been shown to correlate with atherosclerotic plaque development and adverse cardiovascular events (Zheng et al., 2020; Erten, 2021). The molecular mechanism behind this association is yet to be elucidated.

## 4 Inflammation

**FIGURE 4:**
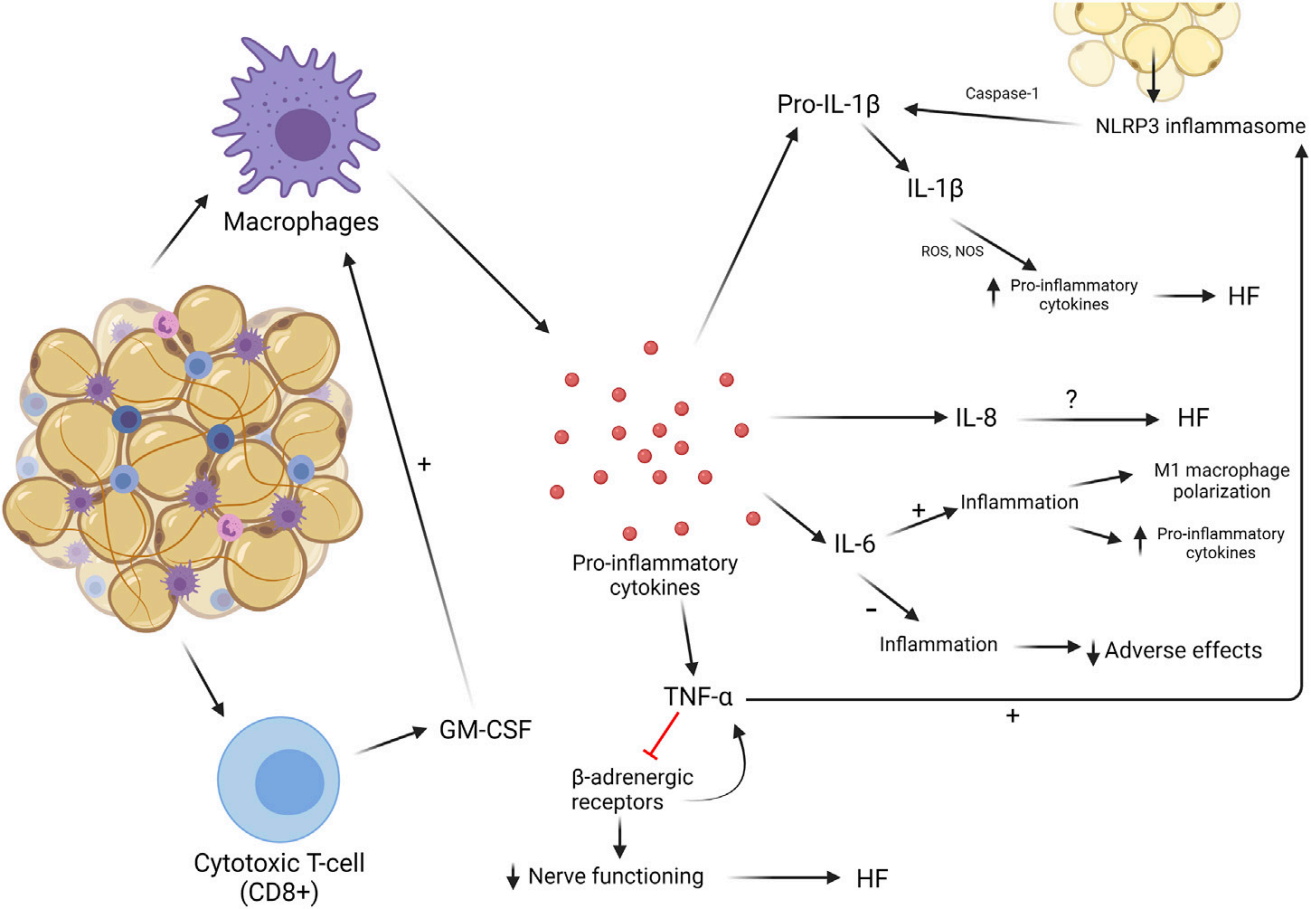
Inflammatory processes in AT. Chronic low-grade AT inflammation involves the recruitment of macrophages and CD8^+^ T cells, which secrete cytokines. This can impact the heart in multiple ways.

Local and systemic inflammation are hallmarks of cardiovascular and metabolic diseases, often associated with a worse prognosis. Whilst some inflammation is reparative in the heart—such as immediate remodeling following myocardial injury—excess and chronic infiltration of macrophages, monocyte activation, and exposure to pro-inflammatory cytokines induce pathological remodeling of the heart (Halade and Lee, 2022). An outline of how AT immune cell activity can contribute to fat–heart crosstalk, directly and indirectly, is depicted in Figure 4.

### 4.1 Leukocytes

#### 4.1.1 Macrophages

Macrophages are resident in both healthy and dysregulated AT (Davies et al., 2013). The relative proportion of macrophages present in AT is in direct proportion to the abundance of AT in the body (Weisberg et al., 2003). In healthy AT in lean individuals, macrophages express an anti-inflammatory phenotype called M2, which helps metabolic regulation and suppression of inflammatory cytokines. In obesity, this phenotype switches toward a pro-inflammatory state, M1, producing a wide array of inflammatory cytokines. This transition is believed to be a slow process, but when no changes are made to the amount of AT in obesity, a vicious circle of macrophage polarization and an increasing inflammatory landscape develop. When this occurs, more macrophages will change from M2 to M1 (Suganami et al., 2005; Lumeng et al., 2007). Cytokines that are most abundantly released by M1 macrophages are IL-6, TNF-α, and IL-1. This increased release attracts more macrophages and other leukocytes toward the site of inflammation, elevating the concentration of macrophages and the production of pro-inflammatory cytokines (Weisberg et al., 2003). These cytokines and their effects on the myocardium and role in CVDs will be discussed later in this review.

#### 4.1.2 CD8 cells

Before macrophages greatly invade AT, they are preceded by cytotoxic T-cells, or CD8 cells. These cells are also resident in AT but not as abundant as macrophages. Nishimura et al. (2009) showed that CD8 cells play a major role in macrophage activation, proliferation, and recruitment by secreting cytokines to induce these effects. CD8 cells can produce GM-CSF, a cytokine that can activate M1 macrophages to induce the pro-inflammatory environment in AT (Yang et al., 2010). Not much is known about their influence in AT-induced inflammation, but they could be an interesting target to potentially attenuate macrophage recruitment and accumulation in AT.

### 4.2 Cytokines and effector mechanisms

#### 4.2.1 NLRP3 inflammasome and IL-1β

NLR family pyrin domain containing 3 (NLRP3) is a protein that forms a primary component of the inflammasome upon stimulation by pathophysiological triggers in damaged or infected tissues (Broz and Dixit, 2016). Studies have reported that obesity can induce the formation of the NLRP3 inflammasome, sustaining the inflammatory environment often noticed in AT in obesity. The NLRP3 inflammasome has the ability to sense obesity-induced danger signals, leading to cardiac remodeling (Vandanmagsar et al., 2011; Sokolova et al., 2019). These signals and NLRP3 inflammasome production are more prevalent in VAT than in SAT. The inflammasome stimulates an enzyme called caspase-1, which is involved in the cleavage of pro-inflammatory cytokines into their active states, mainly IL-1β and IL-18 (Koenen et al., 2011; Vandanmagsar et al., 2011). Stimulation of the IL-1 receptor by IL-1β induces transcriptional and translational alterations in sarcoplasmic/endoplasmic reticulum calcium-ATPase (SERCA) and overexpression of nitric oxide synthases (NOSs), increasing the production of pro-inflammatory cytokines in the myocardium and maintaining a high level of inflammation in both AT and cardiac tissue. When cardiomyocytes were treated with IL-1β, contractile dysfunction was promoted and LV contractility reserve was reduced, leading to HF (Segiet et al., 2019). Cardiac injury stimulated the ROS production and release of mitochondrial (mt) DNA from cardiomyocytes, where ROS stimulates the production of precursor IL-1β and IL-18 by paracrine and endocrine signaling activation of NF-kB and mtDNA stimulates the assembly of the NLRP3 inflammasome. Together, IL-1β and IL-18 were cleaved and activated to stimulate inflammation (Jahng et al., 2016). The NLRP3 inflammasome plays a significant role in a number of cardiomyopathies, including inflammatory cardiomyopathy and myocarditis, both often resulting in HF. The inflammasome makes for an attractive therapeutic target for inflammation-associated syndromes, including obesity-driven cardiometabolic disease (Wang et al., 2020; Li et al., 2021).

#### 4.2.2 IL-8

Another interleukin, IL-8, is also produced by AT and has adverse effects on the myocardium (Nymo et al., 2014; Segiet et al., 2019). VAT produces more IL-8 compared to SAT but not all IL-8 comes from AT in obesity. Nevertheless, AT contributes to the accumulation of this IL in cardiomyocytes (Damås et al., 2000). However, how IL-8 exhibits its influence and how it can act as a therapeutic marker is yet to be investigated.

#### 4.2.3 IL-6

IL-6 is a cytokine that can be both anti-inflammatory and pro-inflammatory. The distinguishing factor as to its functionality is determined by the way it is produced and by what tissue. Trans-signaling differs from classical signaling by the use of gp130, while classical signaling uses the IL-6 receptor. Trans-signaling of IL-6 is pro-inflammatory, leading to M1 macrophage polarization and accumulation, and there is evidence that adipocytes produce IL-6 through this type of signaling (Han et al., 2020). An original study was performed using mouse VAT, but now results of IL-6 in SAT in humans show similar effects—macrophage accumulation and production of pro-inflammatory cytokines (Sindhu et al., 2015). Levels of IL-6 and its receptor were elevated in people suffering from obesity (Segiet et al., 2019). Whitham et al. (2019) investigated how much cytokine production in AT is involved in the concentration of IL-6 in mice. They found that 40% of all IL-6 was AT-derived, and VAT produced more IL-6 than SAT. This suggests that at least 40% of the total amount of produced IL-6 occurs through trans-signaling and, therefore, will express pro-inflammatory properties (Jones and Jenkins, 2018; Whitham et al., 2019). The more chronic the production of IL-6, the more harmful its effects. In patients with end-stage HF, IL-6 was highly upregulated (Segiet et al., 2019). Furthermore, a rat model used by Chen et al. (2022) mimicked HFpEF and showed that levels of IL-6 were increased in these animals, contributing to increased lipolysis and showing the damaging effects of IL-6.

IL-6 contributes to the pro-inflammatory mechanism as a whole by mobilizing M1 macrophages and inducing harmful effects on the myocardium. Its influence is thought to be greater than what is currently known. Its utility as a biomarker for diagnosis and prognosis for cardiac diseases, including congestive heart failure, has been evaluated (Yan et al., 2010; Markousis-Mavrogenis et al., 2019).

#### 4.2.4 TNF-α

As previously mentioned, TNF-α is predominantly produced by M1 macrophages. Since M1 macrophages are abundant in AT, circulating and local TNF-α is highly upregulated in people with obesity, with adverse cardiac effects (Hotamisligil et al., 1995). Increased TNF-α signaling in the heart contributes to HF. It inhibits β-adrenergic receptors, resulting in a decreased sensitivity to changes in cardiac demand. This inhibition then activates a cascade where more TNF-α is produced, eventually leading to HF (Schumacher and Naga Prasad, 2018). TNF-α has the ability to induce the assembly of the NLRP3 inflammasome and modulate its activity. The activity of caspase-1 is highly increased and, therefore, the concentration of IL-1β as well, leading to adverse effects on the heart (Liddle et al., 2020). TNF-α has also been recognized as a potential biomarker for prognosis of HF, as its elevation in concentration showed the worst cardiovascular outcomes (Cohen et al., 2020).

A number of other potent inflammatory mediators that are known to mediate cardiac biology, especially in the context of disease, such as IFN gamma and TGF superfamily members, are also expressed in AT (Lee, 2018; Bradley et al., 2022). However, their role as paracrine mediators between AT and the heart in health and disease is generally not well established. One study looked at the role of cardiac activation of TGF-β1 on adipocyte biology in the context of a high-fat diet. Whilst circulating levels of TGF-β1 were very modestly increased in mice fed a high-fat diet compared with WT mice, the direct effect of cardiac-derived TGF-β1 vs. adipose-derived TGF-β1 is not fully dissociated (Longenecker et al., 2021).

## 5 Discussion

The basis of obesity-associated cardiac disease has long been noted, but the underlying causative pathological fat–heart crosstalk is poorly understood. Crosstalk between the heart and AT is extensive and has many different actors that play a role. The obesity pandemic is not looking at calming any time soon, and there is an urgent unmet need for risk factor-tailored therapeutics to reduce mortality rates due to HF. Adipokines have been interesting targets for many years as they heavily influence the myocardium. Adiponectin is highly expressed and produced in AT and exhibits a cardioprotective purpose. It can decrease the level of inflammation and act as an antioxidant to prevent DNA damage in cardiac cells (Wang et al., 2009; Bobbert et al., 2011). Omentin-1 is also cardioprotective but has a different area of focus, acting as an anti-fibrotic adipokine and suppressing inflammation (Chen et al., 2020; Zhou et al., 2020). A recent addition to the adipokine family is vaspin, protecting the myocardium by reducing TNF-α-induced apoptosis and inhibiting the NLRP3 inflammasome (Ke et al., 2018; Li et al., 2019). All these adipokines are cardioprotective and can restore the damaged cardiomyocytes to a healthy function, using multiple methods of action and can therefore be used in different scenarios and with different co-morbidities that lead to HF. Each patient could get a therapeutic strategy that would suit their situation, showing their potential in the future of personalized medicines. There are other adipokines that are pathogenic in heart function. Resistin is produced by macrophages, whose numbers are increased in AT, and directly influences the myocardium by promoting pro-inflammatory cytokine production and inducing hypertrophy (Patel et al., 2003; Park and Ahima, 2013). Resistin is an adipokine that is not well understood like adiponectin but could potentially be targeted to inhibit its function and therefore decrease the inflammatory environment. Macrophages are accumulated in AT and even higher concentrations exist in AT of obese patients. They secrete pro-inflammatory cytokines that induce inflammation of AT and the myocardium. They are an interesting cell to target as it is responsible for cytokine production but even more due to its multiple roles in crosstalk. These are production of resistin, a pathogenic adipokine, and pro-inflammatory cytokines like TNF-α, IL-6, and IL-1, changing the phenotype of M2 macrophages to the M1 phenotype, which is pro-inflammatory (Suganami et al., 2005; Lumeng et al., 2007).

Further recent insights in fat–heart crosstalk include those *via* microRNAs. A wide array of miRs have been reported to have pathogenic influence on the myocardium, but there are also miRs that are reported to be beneficial in maintaining a healthy heart. This is a novel field in research but it has great potential to become therapeutic targets, since inhibition of these miRs could lead to better patient outcomes. Currently, many of these studies remain at the pre-clinical stage, and further research studies are greatly needed to understand their working mechanisms and potential as therapeutic targets or options to improve cardiac health in obesity in humans.

With the recent acceleration in depth and affordability of multi-omics sequencing technology, overlaying of complementary datasets from fat and the heart, such as transcriptomics data from tissues accompanied by secretome analysis at the level of protein and non-coding RNAs, would provide a wide view on how these two organs communicate in health and disease. Moreover, transcriptomics analysis at the level of the single cell in tissues would provide information as to how different cell types remodel in pathology in order to produce a cardio-detrimental secretome.

To conclude, there is a great unmet need in translational science in understanding how excess AT in obesity severely increases the risk of CVDs. Interception of disease-causing signals between the two organs could provide a new therapeutic axis to reduce the burden of CVDs.
